# Supplementary material for: Idiopathic Plasmacytic Lymphadenopathy Forms an Independent Subtype of Idiopathic Multicentric Castleman Disease
Source: Int J Mol Sci. 2022 Sep 7;23(18):10301. doi: 10.3390/ijms231810301 (PMC9499480; doi:10.3390/ijms231810301)
Supplement: Supplementary file 1 [file ijms-23-10301-s001.zip › ijms-1871779-supplementary.pdf]

Supplementary Table S1. Sites of lymphadenopathy and biopsy sites in iMCD-NOS cases.

Subtype	Age/Sex	Biopsy site	Site of lymphadenopathy	Extranodal lesion
IPL	47/M	inguinal	mediastinum, hilar, inguinal	multiple lung nodules
	35/F	cervical	cervical, supraclavicular fossa, axillary, para-aortic, mesenterium, inguinal	
	55/M	inguinal	mediastinum, inguinal	multiple lung nodules
	62/M	-*	systemic, unspecified *	multiple lung nodules, kidney
	55/F	inguinal	systemic, unspecified *	
	76/M	cervical	cervical, axillary, inguinal	skin
	61/F	axillary	systemic, unspecified *	
	37/M	-*	cervical, axillary, abdominal	
	39/M	inguinal	systemic, unspecified *	multiple lung nodules
	49/F	mediastinum	infraclavicular, mediastinum, axillary, inguinal	
	48/F	axillary	axillary	multiple lung nodules
	59/M	cervical	cervical, axillary, abdominal, inguinal	multiple lung nodules, skin
	64/M	inguinal	cervical, axillary, mesenterium, pelvis, inguinal	
	70/M	cervical	cervical, supraclavicular fossa, mediastinum, axillary, para-aortic	
	54/F	cervical	cervical, axillary	multiple lung nodules
	65/M	cervical	systemic, unspecified *	
	62/F	cervical	cervical (bilateral, multiple)	tonsil swelling
	37/M	inguinal	axillary, inguinal	splenomegaly
	55/F	-*	cervical, supraclavicular fossa, mediastinum, axillary, para-aortic, abdominal, inguinal	
	52/F	axillary	mediastinum, hilar, axillary	multiple lung nodules
	74/F	mediastinum	mediastinum, abdominal	hepatosplenomegaly
	34/M	mediastinum	cervical, mediastinum, hilar	
	57/M	inguinal	cervical, axillary, para-aortic, iliac, inguinal	
	59/M	cervical	cervical, axillary	
	52/F	inguinal	cervical, para-aortic, inguinal	
	70/M	cervical	cervical, mediastinum, para-aortic,	
	43/M	inguinal	supraclavicular fossa, inguinal	
	41/M	cervical	cervical, supraclavicular, axillary, inguinal,	skin
	49/M	axillary	cervical, supraclavicular, mediastinum, axillary, inguinal	hepatosplenomegaly, kidney
	48/F	axillary	axillary, para-aortic, abdominal	hepatosplenomegaly
	38/F	inguinal	cervical, mediastinum, inguinal	multiple lung nodules, splenomegaly, skin
	76/M	cervical	cervical, subclavicular, axillary, para-aortic, pelvis, inguinal	
	67/M	inguinal	axillary, inguinal	
	72/M	cervical	cervical (bilateral, multiple)	
non-IPL	70/M	supraclavicular fossa	supraclavicular, mediastinum	
	52/F	axillary	cervical, axillary, inguinal	kidney
	89/M	para-aortic (needle biopsy)	mediastinum, retroperitoneum (multiple)	
	73/F	axillary	mediastinum, axillary	
	49/F	axillary	systemic, unspecified *	multiple lung nodules, skin
	32/M	cervical	systemic, unspecified *	hepatosplenomegaly
	49/F	cervical	systemic, unspecified *	kidney, skin
	49/F	cervical	systemic, unspecified *	kidney

Multiple lymphadenopathies were noted in all cases. Sampling site was unknown in 3 cases of IPL group. All cases had excisional biopsy except for one non-IPL case who had a core needle biopsy. * Details unknown.
